# Phenotypic high-throughput screening identifies aryl hydrocarbon receptor agonism as common inhibitor of toxin-induced retinal pigment epithelium cell death

**DOI:** 10.1371/journal.pone.0301239

**Published:** 2024-04-18

**Authors:** Joshua Schustak, Hongwei Han, Kyle Bond, Qian Huang, Magali Saint-Geniez, Yi Bao

**Affiliations:** Department of Ophthalmology, BioMedical Research, Novartis, Cambridge, Massachusetts, United States of America; Charite Universitatsmedizin Berlin, GERMANY

## Abstract

The retinal pigment epithelium (RPE) is essential to maintain retinal function, and RPE cell death represents a key pathogenic stage in the progression of several blinding ocular diseases, including age-related macular degeneration (AMD). To identify pathways and compounds able to prevent RPE cell death, we developed a phenotypic screening pipeline utilizing a compound library and high-throughput screening compatible assays on the human RPE cell line, ARPE-19, in response to different disease relevant cytotoxic stimuli. We show that the metabolic by-product of the visual cycle all-trans-retinal (atRAL) induces RPE apoptosis, while the lipid peroxidation by-product 4-hydroxynonenal (4-HNE) promotes necrotic cell death. Using these distinct stimuli for screening, we identified agonists of the aryl hydrocarbon receptor (AhR) as a consensus target able to prevent both atRAL mediated apoptosis and 4-HNE-induced necrotic cell death. This works serves as a framework for future studies dedicated to screening for inhibitors of cell death, as well as support for the discussion of AhR agonism in RPE pathology.

## Introduction

The retinal pigment epithelium (RPE) forms a unique monolayer at the outermost region of the retina, interacting with photoreceptors on its apical side, and with Bruch’s membrane and the choriocapillaris on its basal side [1]. RPE cells are essential for supporting the photoreceptors, maintaining visual function, and renewal of visual pigments [2]. However, genetic and environmental factors can cause RPE functional loss and atrophy [3, 4]. RPE dysfunction and death play central roles in severe ocular degenerative diseases, such as age-related macular degeneration (AMD), Stargardt disease, and retinitis pigmentosa [5, 6]. In fact, death of RPE is a clinical hallmark of AMD progression to advanced stages [6]. Unfortunately, the intrinsic and exogenous drivers of RPE cell death remain controversial [7].

Deficiency in key enzymes and/or exposure to excessive light leads to disrupted clearance and accumulation of detrimental intermediate metabolites generated by the visual cycle, such as all-trans-retinal (atRAL) [5, 8]. atRAL was found to be cytotoxic in both rat and human RPE cells [9–11]. Furthermore, retinopathy was observed in mice lacking proteins critical for atRAL clearance, such as Rdh8 and Abca4 [8, 11, 12].

Oxidative stress caused by cigarette smoking, UV light, ionizing radiation, and diet has also been implicated in RPE degeneration [7]. 4-hydroxynonenal (4-HNE), a toxic byproduct of stress-induced lipid peroxidation, has been shown to be significantly increased in the retina of AMD donors as well as in patient plasma [13], and causes RPE degeneration in vitro [14]. In a mouse model recapitulating a dry AMD-like phenotype, 4-HNE accumulation was associated with RPE degeneration [15].

These RPE stressors can lead to cell death through several mechanisms, including apoptosis and necroptosis [7]. Apoptosis is a form of programmed cell death, which can occur through either intrinsic or extrinsic signaling pathways, leading to degradation of key cellular components in a highly controlled manner. Apoptosis is regulated by the caspase family of proteins including caspase 3/7/8 [16]. In contrast, necroptosis involves the rupture of intracellular organelles and plasma membrane through activation of receptor-interacting protein kinases (RIPKs) [17]. Increased knowledge on cell death mechanisms makes it an attractive target for therapeutic intervention.

The use of caspase inhibitors for the treatment of inflammatory, neurological, and metabolic disease has shown little success, indicating that targeting apoptosis alone is not sufficient [18]. Over the past few years, greater appreciation and consideration has been given to alternative cell death pathways, such as the necrotic type of cell death. Understanding the different types of cell death pathways involved in AMD can provide a new perspective on the factors driving disease progression.

In this study, we defined the specific types of cell death triggered by atRAL and 4-HNE in ARPE-19 cells. We found that atRAL induces apoptosis, whereas 4-HNE induces necrotic cell death. To identify compounds able to prevent RPE cell death from both toxins, we developed a screening pipeline utilizing a CytoTox-Glo assay to identify atRAL-induced cell death inhibitors, followed by a propidium iodide (PI) measurement to assess the capacity to prevent 4-HNE-induced death. The top hit, 2-(4-Amino-3-methylphenyl)benzothiazole (DF 203), a reported aryl hydrocarbon receptor (AhR) agonist, was further validated. This screening platform can quickly identify and discover new pathways and targets modulating toxin-induced RPE cell death.

## Results

### Different retinal stressors induce distinct cell death mechanisms in RPE cells

Light-induced retinoid accumulation associated with visual cycle defects and smoking-induced oxidative stress are two environmental risk factors causing toxic metabolites to accumulate in the retina, especially in RPE, and promote cell death. To mimic this pathological process in vitro, we utilized the toxic metabolites all-trans-retinal (atRAL) to represent retinoid accumulation, and 4-hydroxynonenal (4-HNE) to mimic oxidative stress, on ARPE-19 cells, a human RPE cell line [19]. Toxin exposure induced cell death in both a dose- and time-dependent manner, as previously reported (S1A–S1C Fig) [10, 14].

Interestingly, we observed different morphological changes of ARPE-19 following exposure to cytotoxic doses of atRAL or 4-HNE; atRAL caused cell shrinkage and formation of small high-density cell bodies, characteristic of apoptosis, while 4-HNE triggered minor morphology changes without shrinkage (S1A Fig). We hypothesized that these morphological differences were due to induction of different cell death mechanisms.

**Fig 1:**
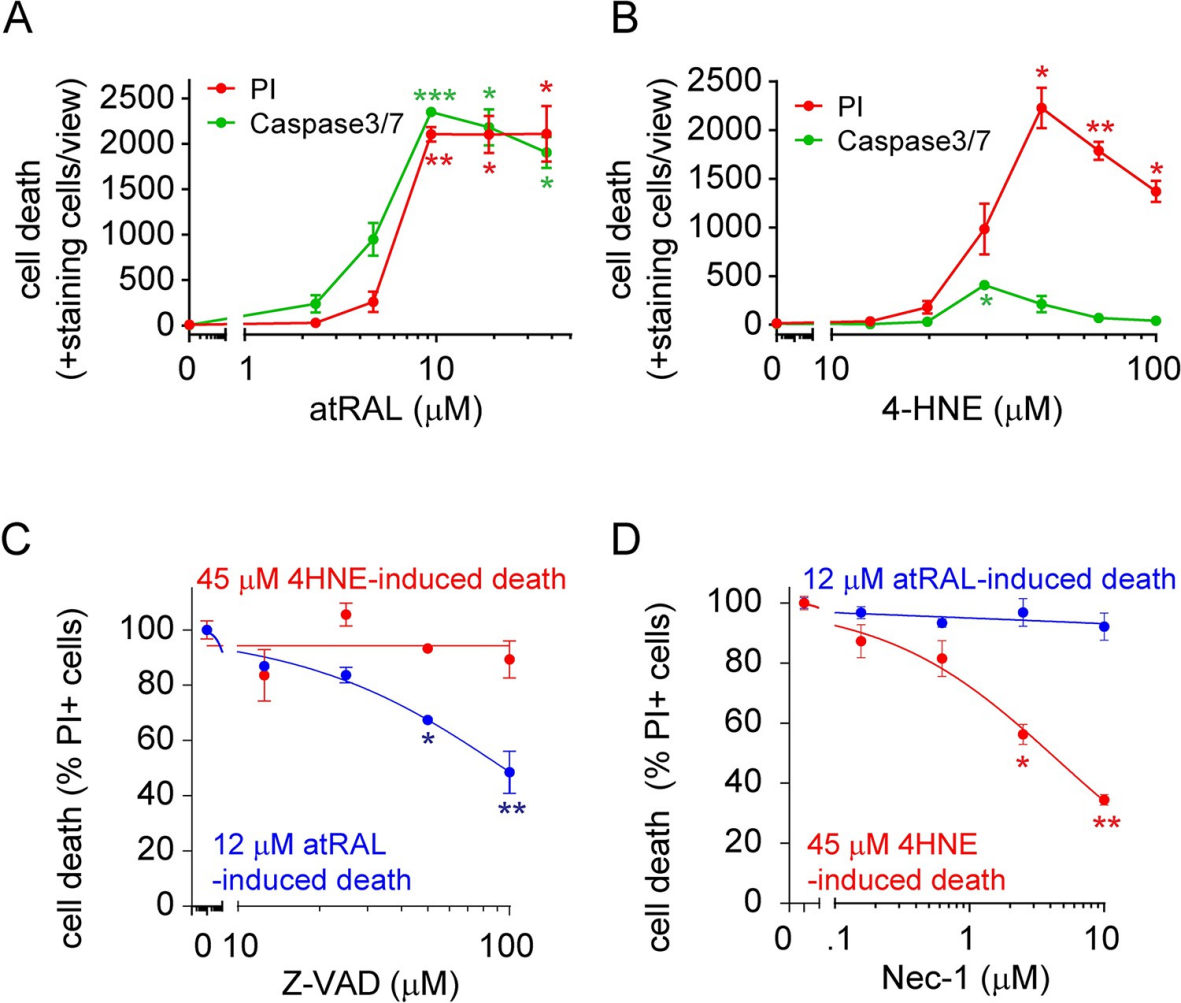
atRAL induces apoptosis while 4-HNE induces necroptosis in ARPE-19 cells. (A-B) Analysis of general cell death (PI) and caspase3/7 mediated apoptosis (CellEvent) following atRAL (A) and 4-HNE (B) treatments. (C-D) Quantification of atRAL- or 4-HNE-mediated ARPE-19 cell death using PI following pretreatment with increasing doses of Z-VAD (C) or Nec-1 (D) before challenge. Each data point represents biological replicates (n = 3–4) with five images captured per replicate and indicated as mean±S.D. Non-parametric Kruskal-Wallis test was applied for statistical analysis. * p<0.05, ** p<0.01 and *** p<0.001 compared to vehicle control.

To determine the type of cell death induced by those toxins, assays designed to discriminate between apoptotic and necrotic cell death were conducted. Caspase-3/7 cleavage is a hallmark of apoptosis [20], so we used CellEvent (Invitrogen), a novel fluorogenic substrate for activated caspase-3/7, to quantify kinetics of apoptosis and compare with PI-positive staining for pan-cell death. Interestingly, atRAL treatment induced near-identical PI and CellEvent Caspase3/7 dose responses (Fig 1A). Conversely, 4-HNE induced PI positivity while CellEvent signal remained low to undetectable (Fig 1B). These results indicate that atRAL promotes an apoptotic type of cell death, while 4-HNE promotes necrotic cell death.

To confirm the mechanisms of toxin-induced cell death in ARPE-19, we assessed cell viability in conjunction with specific cell death inhibitors. The pan-caspase inhibitor Z-VAD, an apoptosis inhibitor [21], was predicted to prevent atRAL induced cell death but not 4-HNE. In contrast, the RIPK1/3 inhibitor Necrostatin-1 (Nec-1), which can block necroptosis [22], was expected to show the inverse. ARPE-19 cells were pretreated with Z-VAD or Nec-1 for 1 h before challenge with atRAL or 4-HNE, and pan-cell death was quantified by PI staining in an IncuCyte. As anticipated, Z-VAD treatment rescued atRAL- but not 4-HNE-induced cell death while Nec-1 inhibited 4-HNE- induced cell death but had no effect of atRAL-treated cells (Fig 1C and 1D).

### Compound screening pipeline developed to discover inhibitors of toxin-induced cell death

**Fig 2:**
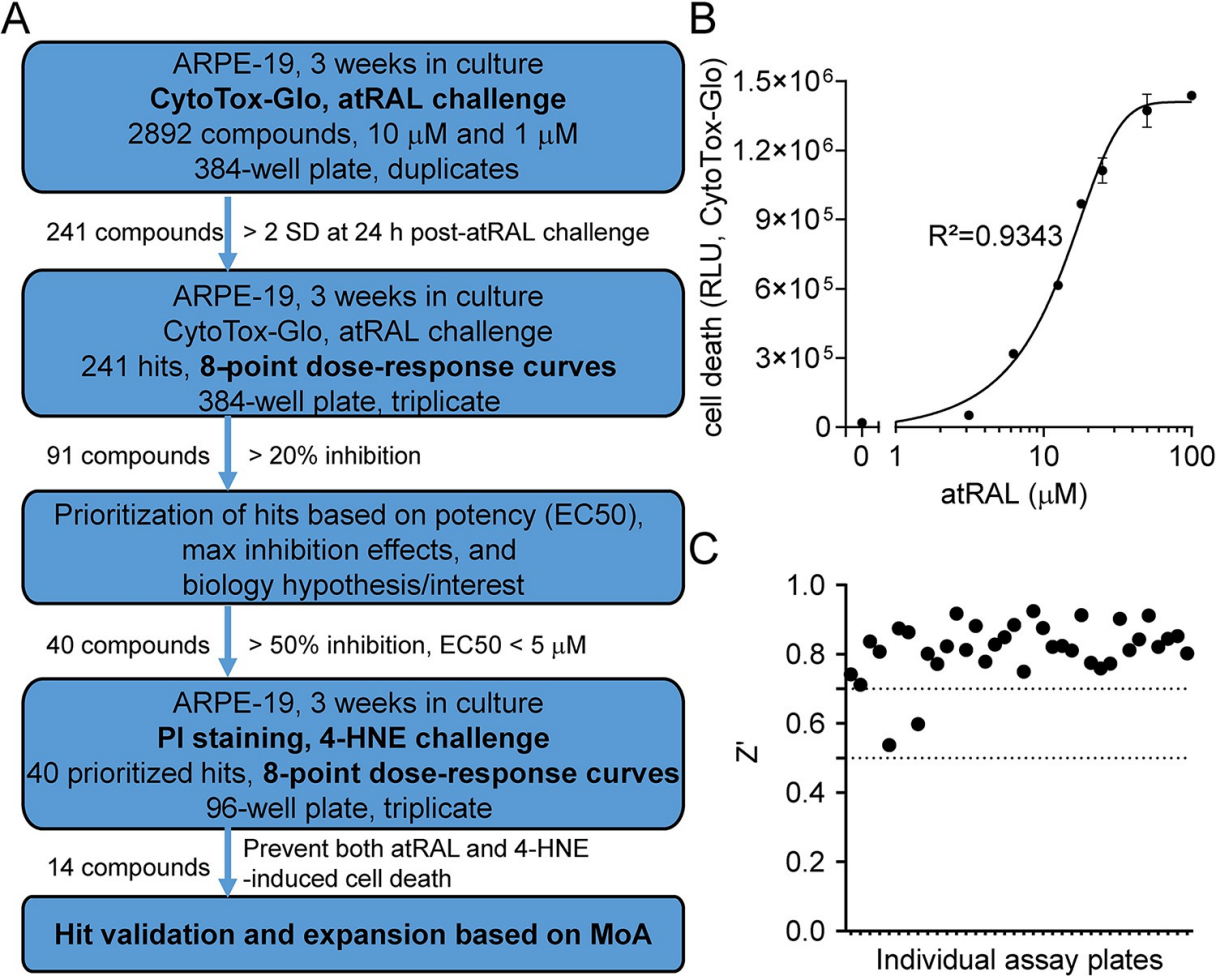
Compound screen identification for prevention of both atRAL and 4-HNE-induced ARPE-19 cell death. (A) Screening flowchart for the study, showing experiment treatment conditions, hit selection criteria and number of compounds passing each step. (B) Dose-dependent atRAL induction of ARPE-19 cell death, detection using CytoTox-Glo (EC50 = 12.0 μM, R2 = 0.9343). (C) Z’ factor for all assay plates was calculated to confirm screening quality based on vehicle control and 18 μM atRAL treatments. Outliers (Z’<0.7) were excluded from further analysis.

AMD is a multifactorial disease, and its progression may not be dependent on any single mechanism of cell death. Preventing atrophy in AMD may be a challenge using approaches targeting a unique cell death pathway, as our findings with Z-VAD and Nec-1 predict. To tackle these challenges, we developed a compound screening strategy aiming to discovering potential treatments that could simultaneously prevent multiple forms of cell death (Fig 2A).

We utilized a compound library of 2,892 compounds, all with known targets [23], to identify pathways able to prevent toxin-induced ARPE-19 cell death (Fig 2A). In brief, atRAL-challenged ARPE-19 cells were treated with compounds and analyzed for cell death. Two compound concentrations (1 and 10 μM) were used to rigorously identify pathways of interest. A luminescence-based high-throughput cytotoxicity assay (CytoTox-Glo), which measures dead cell-released protease activity, was utilized as the primary screening modality (Fig 2B). Z’ factors were evaluated to confirm screening quality. Out of 36 assay plates, two plates had a Z’ below 0.7 and were excluded from further analysis (Fig 2C).

**Fig 3:**
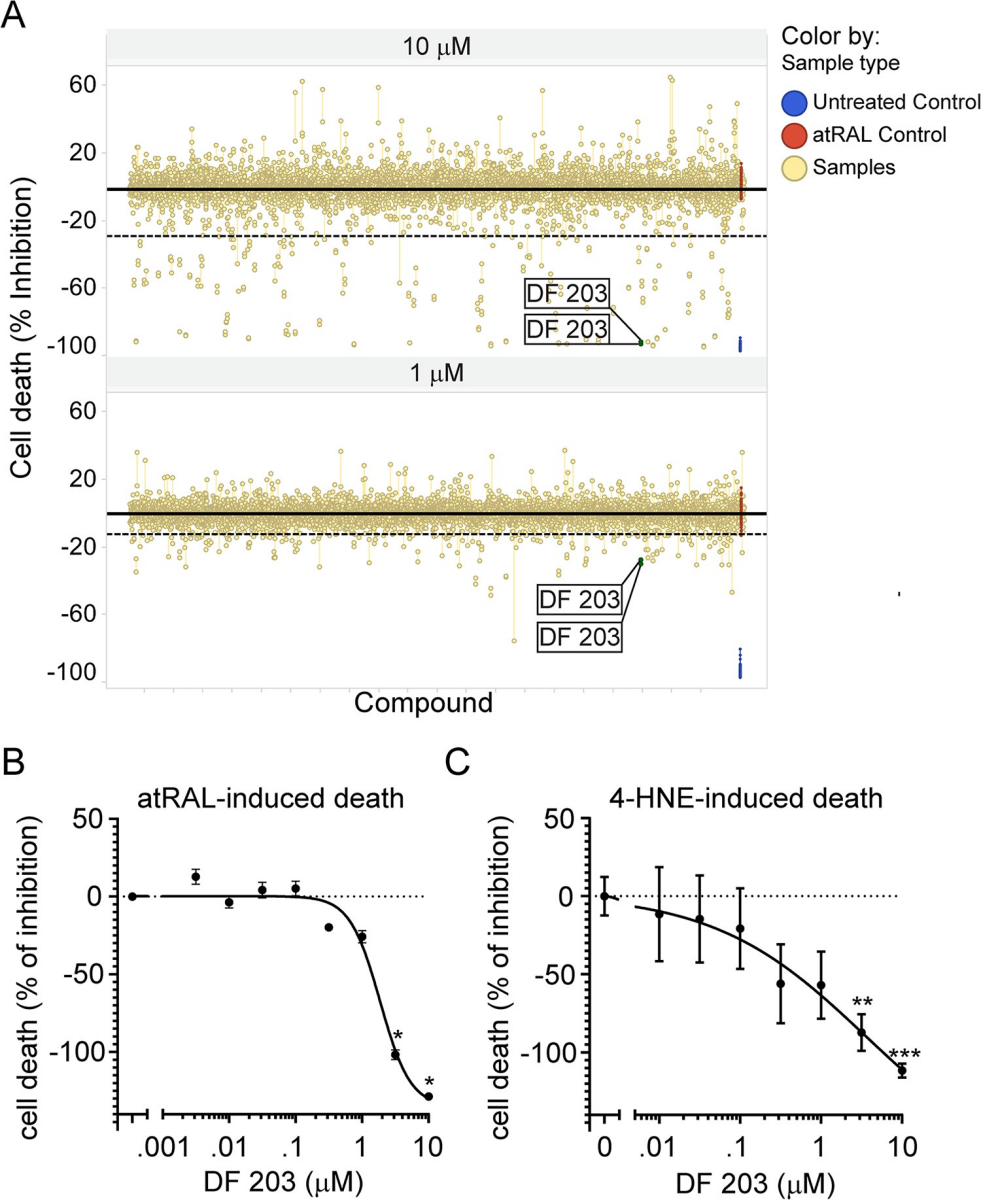
DF 203 identified as target inhibiting atRAL- and 4-HNE-induced ARPE-19 cell death. (A) Percentage inhibition of compounds on atRAL-induced ARPE-19 cell death. atRAL-treated group-only set as 0% inhibition, and untreated group set as 100% inhibition. Dotted line indicates 2xS.D. cutoff limit. The yellow line linked 2-dots replicates. (B) Dose-dependent inhibition of atRAL-mediated cell death of ARPE-19 by DF 203 measured by CytoTox-Glo. (C) Dose-dependent inhibition of 4-HNE mediated cell death of ARPE-19 by DF203 measured by PI. Each data point (B-C) represents biological replicates (n = 3–4), and indicated as mean±S.D.. Non-parametric Kruskal-Wallis test was applied for statistical analysis. * p<0.05, ** p<0.01 and *** p<0.001 compared to vehicle control.

After confirming the screening results using an 8-point dose-response curve on atRAL-induced death, hits were further screened on 4-HNE-induced ARPE-19 cell death, measured by PI staining, to identify compounds that could also prevent 4-HNE induced ARPE-19 cell death (Fig 3A). This secondary screen identified 14 compounds able to prevent both toxic-inducers of ARPE-19 cell death. One hit, DF 203 (2-(4-Amino-3-methylphenyl) benzothiazole), is shown as an example with an 8-point curve dose-response provided for both toxic-inducers (Fig 3B and 3C).

### AhR agonists prevent toxin-induced RPE damage

DF 203 was originally identified as an anti-cancer agent and aryl hydrocarbon receptor (AhR) agonist [24]. Previous reports have shown an important protective role of AhR activation in ocular diseases, including AMD [25]. RPE cells are known to express AhR, and its loss has been shown to cause RPE dysfunction, increase inflammation, and lead to atrophy [26–28].

**Fig 4:**
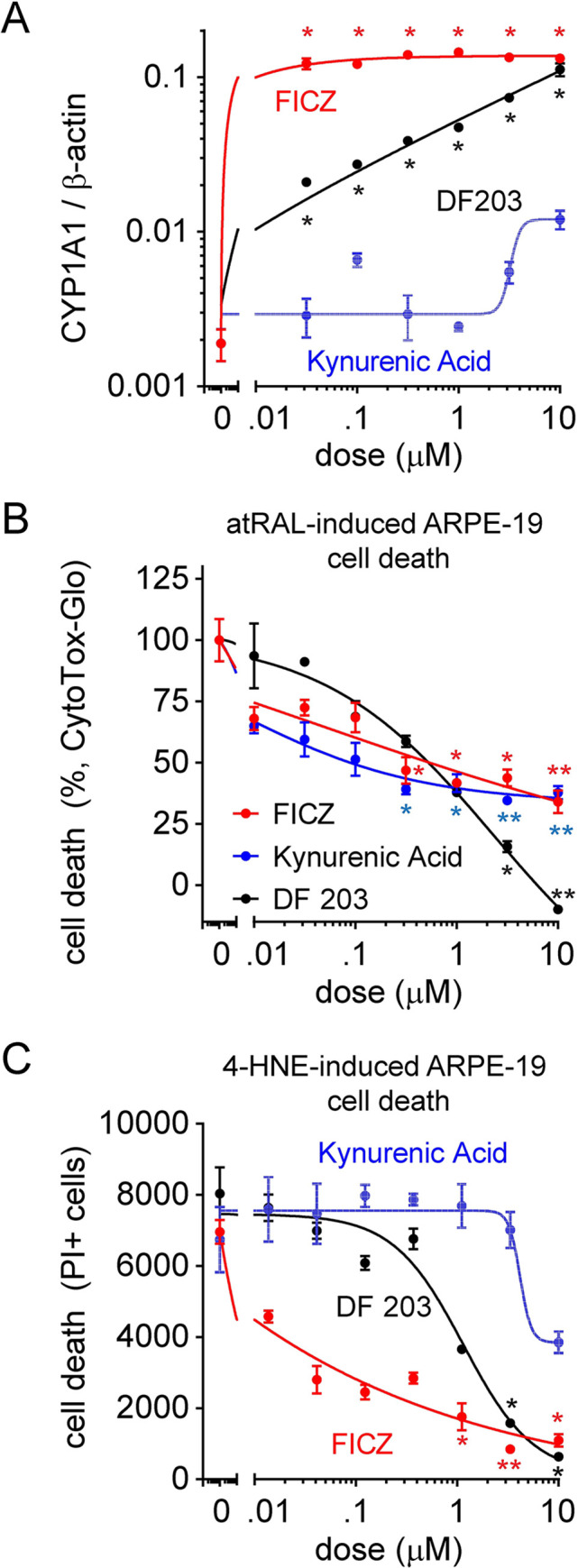
AhR agonists prevent both atRAL and 4-HNE-induced ARPE-19 cell death. (A) Measurement of CYP1A1 transcription in ARPE-19 after dosed treatment with AhR agonists DF 203, FICZ and Kynurenic Acid. CYP1A1 gene expression levels were normalized to β-actin. (B-C) atRAL and 4-HNE mediated cell death inhibition after pre-treatment with AhR agonists, measured by CytoTox-Glo (B) or PI (C). Each data point represents biological replicates (n = 3–4) and indicated as mean±S.D. Non-parametric Kruskal-Wallis test was applied for statistical analysis. * p<0.05 and ** p<0.01 compared to vehicle control.

To evaluate whether AhR activation may act as a general protective mechanism against ARPE-19 cell death induced by toxic metabolites, two additional AhR agonists, FICZ and kynurenic acid, were tested. Classical AhR induction leads to complex formation with Arnt, and translocation to the nucleus to initiate transcription of genes with XRE elements, including the AhR biomarker CYP1A1. We thus measured CYP1A1 transcript levels in ARPE-19 in the presence of AhR agonist treatment to confirm efficacy during toxic challenge [25]. We show that FICZ has potent AhR agonist activity compared to DF 203, while kynurenic acid displayed very weak AhR activation (Fig 4A). All 3 AhR agonists exerted similar protective effect on atRAL-induced cell death (Fig 4B). Importantly, their protective capabilities against 4-HNE cytotoxicity reflected their AhR activity, with FICZ having the strongest effect (Fig 4C). These results indicate a cytoprotective role of AhR activation against both necroptosis and apoptosis, albeit with differing efficacy.

**Fig 5:**
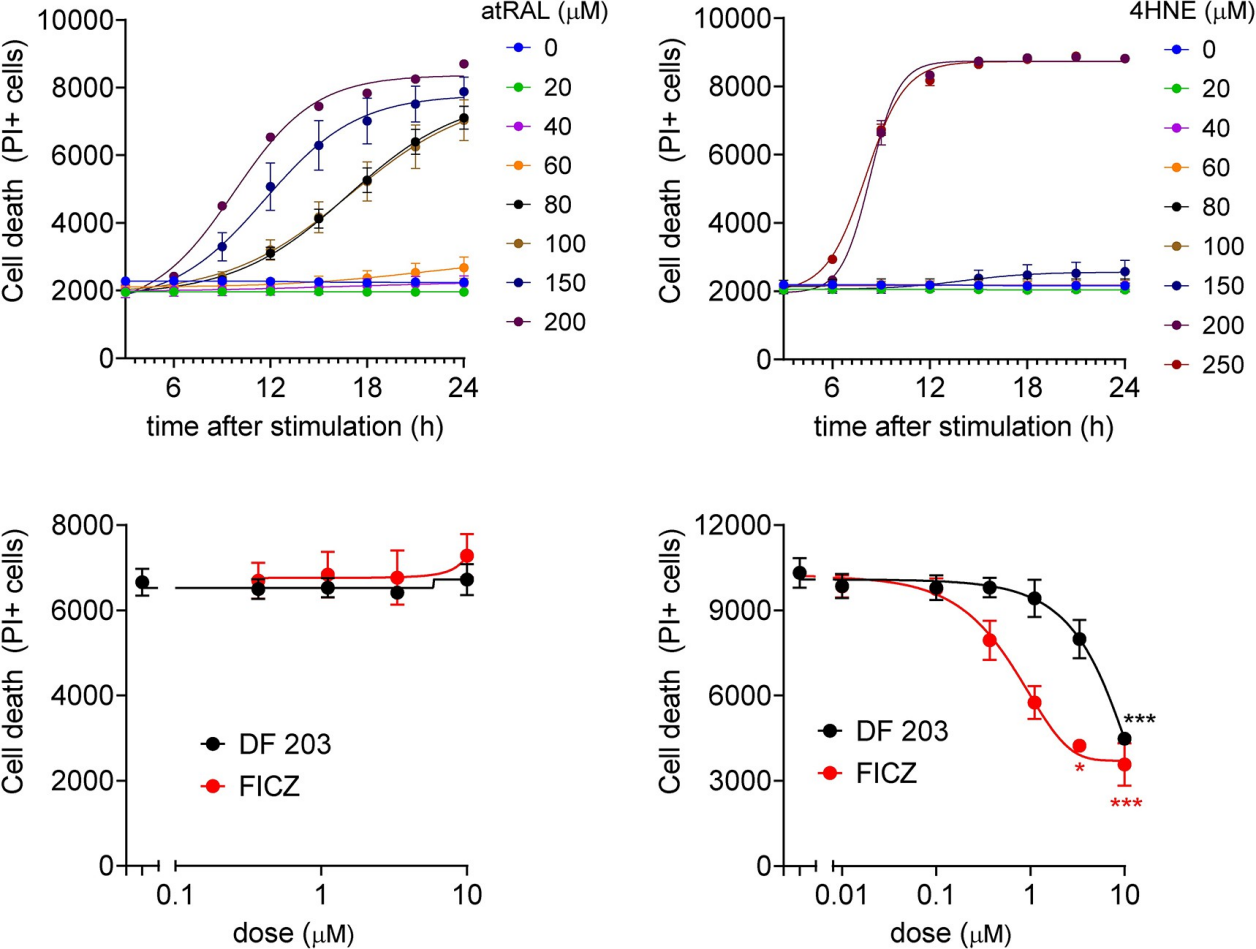
AhR agonists could prevent 4-HNE but not atRAL induced iPS-RPE cell death. (A-B) Analysis of general cell death (PI) following atRAL (A) and 4-HNE (B) treatments. (C-D) AhR agonist effects on atRAL (C) and 4-HNE (D) mediated iPS-RPE cell death, measured by PI staining. Each data point represents biological replicates (n = 3–4) and indicated as mean±S.D. Non-parametric Kruskal-Wallis test was applied for statistical analysis. * p<0.05 and *** p<0.001 compared to vehicle control.

ARPE-19 is a widely used human derived RPE-like cell line, however it has been reported to lack some features of mature RPE, such as pigmentation and RPE65 expression, while retaining some RPE features such as morphology, polarization, and phagocytosis [19]. Due to these limitations, we also utilized iPS-derived RPE to further evaluate AhR agonists on toxin-induced cell death. atRAL and 4-HNE can successfully induce iPS-RPE cell death (Fig 5A and 5B), and AhR agonists could prevent 4-HNE induced iPS-RPE death (Fig 5D). However, surprisingly, AhR agonists did not prevent atRAL induced iPS-RPE death at the dose between 0.1–10 μM (Fig 5C).

## Discussion

RPE cell death has been reported to be the primary contributor to several ocular diseases and disorders, including AMD, Stargardt disease and retinitis pigmentosa. Despite identification of the key drivers of RPE stress, therapies have remained elusive. It is thus critical to better understand the downstream mechanisms resulting in RPE death. Oxidative stress, represented by 4-HNE, and accumulation of toxic visual cycle metabolites, represented by atRAL, are two common triggers of stress-induced RPE death, which may contribute to the pathogenesis of AMD. However, the specific cell death mechanisms induced by these two common cytotoxins are still under debate. Our study showed that atRAL and 4-HNE induced distinct types of cell death of RPE, namely apoptosis and necroptosis, respectively. Similar observations have been previously reported; Cai, B. et al. reported that atRAL can induce apoptosis within the mouse retina [29]. On the other hand, Kaarniranta, K. et al observed necroptotic morphological changes in ARPE-19 exposed to 4-HNE [30]. However, alternative cell death pathways, such as ferroptosis and apoptosis were also reported to be induced after treatment of 4-HNE in RPE [14, 31]. Additionally, unlike atRAL, the cell death induced by 4-HNE exhibited a bell-shaped curve, with a decrease in signal observed at higher doses or longer treatment times (Fig 1B and S1 Fig). We hypothesize that this decrease in signal at higher doses and longer treatment times may be attributed to the reagents and compounds used in our study. Specifically, nuclear membranes become permeable in dead cells, allowing PI to stain nuclei. However, in necrotic cell death, cells may undergo a different phenomenon at the final stage, in which they "explode" and release all intracellular components, ultimately resulting in a loss of signal. This further supports our conclusion that 4-HNE induces necrotic cell death in RPE cells. Furthermore, as a highly reactive chemical, 4-HNE may damage the PI and caspase 3 reagents at high doses, resulting in a loss of fluorescence signal. Further research to fully understand the mechanisms of cell death of RPE cells and the potential crosstalk between various cell death pathway could provide more hints on potential therapeutic targets.

Given the selectivity of the types of RPE cell death induced by these two stressors, we employed a screening approach to identify novel protective compounds and associated pathways. Our study, which identified 14 hits from 2892 compounds (hit rate 0.48%), is among the few conducting high through-put screening for RPE cell death [32, 33]. The advantage of utilizing two distinct RPE degeneration inducers enabled compound hits from our screening assay to have a broader effect targeting cell death. Such “pan-cell death” inhibitors would be potentially effective for a wider range of pathogenic drivers and disease conditions or stages.

**Fig 6:**
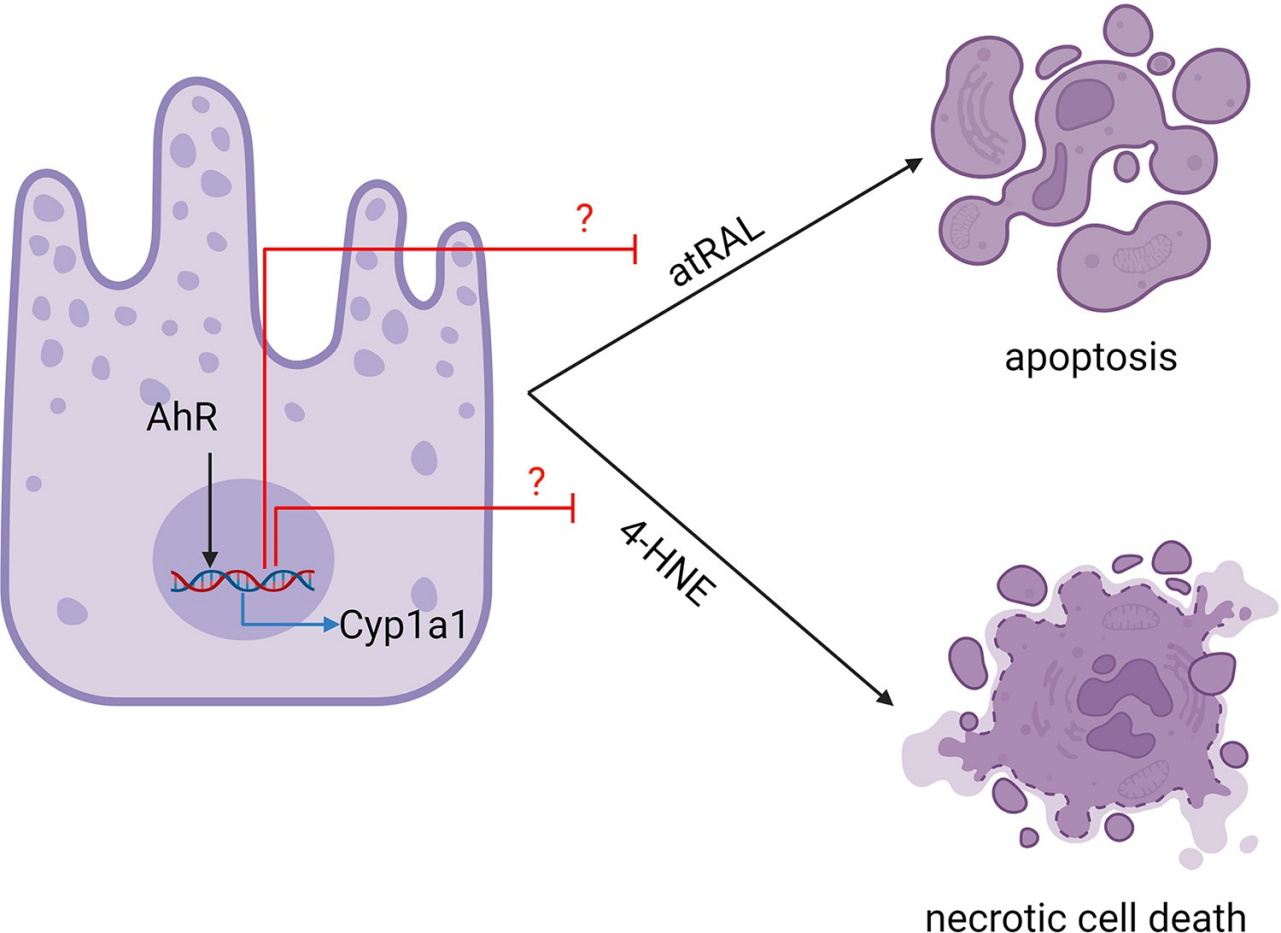
Schematic mechanism of AhR agonism preventing toxin-induced RPE death. Various toxins can induce different types of cell death. In ARPE-19 cells, all-trans retinal (atRAL) induces apoptosis, while 4-hydroxynonenal (4-HNE) induces necrotic cell death. Activation of the aryl hydrocarbon receptor (AhR) leads to the upregulation of several downstream signaling pathways, which may include enzymes and other factors that can prevent cell death caused by these toxins. Figure created with BioRender.com.

Our results suggest that the AhR pathway is essential for promoting health and survival of RPE cells in response to toxin induced cell death. A schematic diagram (Fig 6) was included to provide a concise summary of the proposed mechanism of action. AhR is well known for its role in regulating toxicity [25]. It is a transcription factor that senses endogenous (such as oxygen tension or redox potential) and exogenous factors (such as environmental toxins) and mediates adaptive response to stress via downstream transcriptional changes. Knock-out of AhR in mice can lead to age-related macular degeneration-like pathology [27, 34], suggesting a role for AhR in protecting RPE cells from chronic environmental stress. It has been shown that 2AI, an AhR ligand, can protect RPE cells from 4-HNE-mediated stress, and light-mediated retinal degeneration in mice [26]. The protective effect of 2AI in this model is proposed to rely on a crosstalk between AhR and NRF2 pathways leading to the induction of antioxidant genes [26].

It is interesting to note that in our study, the capacity of DF 203, FICZ, and kynurenic acid to decrease 4-HNE-induced cell death correlated to their ability to activate AhR. But this is not the case for atRAL-induced cell death. This may point to a differential mechanism of agonist-dependent AhR protection between atRAL- and 4-HNE stimulated RPE cell death. Alternatively, this may be due to off-target effects of these compounds. For example, FICZ is known to exert additional biological effects when compared to other members from the same group of tryptophan-derived AhR ligands such as KYN and KYNA, although they share similar activity towards AhR. For example, FICZ has been shown to stimulate cell growth at low concentrations but promote activation of apoptosis via a mitochondrial-dependent pathway [35].Thus, the mechanism of action of FICZ might not be restricted to AhR [28]. DF 203, however, only displayed anti-proliferative effects, which potentially is attributed to emergence and subsequent degradation of unstable biotransformation products [36]. Although further studies are needed to define the specific mechanism of activity of FICZ, DF 203 and kynurenic acid towards atRAL-induced ARPE-19 death, taking into consideration the obtained results, AhR agonism undoubtedly protected ARPE-19 from atRAL stimulated death.

To strengthen our findings, we conducted an evaluation of the effects of AhR agonists on iPS-RPE cells. Interestingly, while atRAL and 4-HNE are also cytotoxic to iPS-RPE cells, these compounds required significantly higher doses to induce complete cell death (Fig 5A and 5B). The increased resistance to toxins in iPS-RPE cells may be attributed to the presence of a more robust anti-oxidative capacity and a stronger barrier function. These factors combine to enhance the overall resistance of iPS-RPE cells compared to ARPE-19 cells when exposed to toxins. Notably, AhR agonists were found to successfully prevent 4-HNE-induced cell death in iPS-RPE cells (Fig 5D), aligning with our results in ARPE-19 cells. However, it was surprising to find that AhR agonists were unable to prevent atRAL-induced cell death in iPS-RPE cells at doses ranging from 0.3 to 10 μM (Fig 5C). This discrepancy can be attributed to several potential factors. First, as mentioned above, iPS-RPE anti-oxidant capacity may be more powerful than ARPE-19 leading to reduced formation of toxic metabolites under similar toxin exposure. Second, iPS-RPE cells may have a higher barrier function, causing differences in cellular drug distribution between ARPE-19 and iPS-RPE cells, and requiring a much higher dose to achieve similar effects, potentially risking toxicity. Finally, the types of cell death induced by atRAL may differ between ARPE-19 and iPS-RPE cells. Indeed, we conducted an evaluation of key apoptotic-related genes in both ARPE-19 and iPS-RPE cells and found higher expression of Fas, Bax, and Casp8 in ARPE-19 cells compared to iPS-RPE cells (S2 Fig), thereby suggesting that iPS-RPE cells may be less sensitive to apoptotic induction. Therefore, high dose atRAL may promote iPS-RPE cell death via a non-apoptotic pathway and, consequently, AhR agonists may not be effective in preventing this alternative cell death. Moreover, as previously discussed, it is possible that AhR agonism work through alternative pathways to prevent atRAL-induced cell death in ARPE-19 cells, and these mechanisms may also apply to iPS-RPE cells, resulting in reduced effectiveness in preventing atRAL-induced cell death. The precise mechanism of action and the distinctions between ARPE-19 and iPS-RPE cells present intriguing avenues for further investigation.

Our study identified AhR agonism as a potential therapeutic approach for blinding ocular diseases associated with RPE degeneration and death. AhR, an important immunomodulator, also has strong potential for therapeutic intervention in multiple inflammatory diseases such as sclerosis and rheumatoid arthritis [37]. One challenge of using AhR agonists is safety, as many agonists are reported associated with severe side effects when used over a long-term [38]. Fortunately, an AhR agonist, Tapinarof cream 1%, has been approved in the USA to treat plaque psoriasis in adults, and is under investigation for the treatment of atopic dermatitis [39], indicating that the development of AhR agonists with fewer side effects could be achieved.

In conclusion, this work indicates there is therapeutic potential of expanding the use AhR agonism to the treatment of ocular diseases such as AMD. Additionally, we further propose utilizing screening strategies that encompass multifactorial disease features to bring greater confidence in target selection.

## Materials and methods

### Cell culture

ARPE-19 was purchased from ATCC (Manassas, VA; CRL-2302) and cultured in DMEM/F12 (Gibco, Carlsbad, CA) supplemented with 10% heat-inactivated FBS (Sigma-Aldrich, St. Louis, MO) and 1% penicillin/streptomycin (Gibco) at 37°C and 5% CO2. Cells were seeded at high density and maintained for at least 3 weeks to form mature monolayers. The experiments were carried out on passages 8–15.

iCell-RPE (Donor #01279), an iPS-derived RPE cell line, was purchased from FUJIFILM Cellular Dynamics (Madison, WI). Cells were expanded in RtEBM medium supplemented with 2% FBS at 37°C and 5% CO2. Cells were maintained at least 3 weeks post-seeding in serum free RtEBM media to form mature monolayers.

### Reagents

All-trans-retinal (atRAL) (TRC, Canada), 4-Hydroxynonenal (4-HNE) (Sigma, Burlington, MA), 2-(4-Amino-3-methylphenyl)benzothiazole (DF 203) (eMolecules, San Diego, CA), Z-VAD (R&D, Minneapolis, MN), Necrostatin-1 (Biovision, Waltham, MA), 5,11-Dihydroindolo[3,2-b]carbazole-6-carboxaldehyde (FICZ), and kynurenic acid (Tocris, Minneapolis, MN) were used in this study. atRAL was stored and manipulated with limited light exposure.

### Measuring cell death

Cell death was measured using CytoTox-Glo cytotoxicity assay (Promega) according to manufacturer’s protocol. Luminescent signal was measured using microplate reader Infinite M1000 (TECAN, Switzerland).

Propidium iodide (PI) and CellEvent Caspase-3/7 were used for dead cell detection (Thermo Fisher, Waltham, MA) following manufacturer’s protocols. Fluorescent images were recorded over time using IncuCyte (Sartorius).

### Primary screening and hit confirmation

2,892 compounds (Novartis mechanism-of-action box) with publicly available chemical structures against known targets was utilized [23]. Compounds were assayed in duplicate at 1 and 10 μM. Z’ was calculated for each assay plate as a quality control measure, with Z’<0.7 excluded from further analysis. Compounds that caused a decrease in atRAL-induced cytotoxicity >2 times of standard deviations from the mean for each concentration at 24 h were classified as a hit.

Preliminary hits were confirmed with an 8-point concentration curve (3.3-fold dilution starting from 10 μM) in duplicate in the CytoTox-Glo assay. Data was normalized relative to negative assay control (0.1% DMSO), and EC50 values were calculated. Hits with EC50 values < 5 μM and max inhibition > 50% were selected to secondary confirmation for 4-HNE induced death measured by PI staining with 8-point dose-response.

### Real-Time RT-PCR

Total mRNA was isolated using TurboCapture 96 mRNA Kit (Qiagen) and reverse-transcribed using High-Capacity cDNA Reverse Transcription Kit (Applied Biosystems, Foster City, CA). Real-Time PCR was performed using Taqman probes. β-actin was used as the control (Applied Biosystems). Reactions were run with Taqman Universal PCR Master Mix on the ViiA7 system (Applied Biosystems) according to the manufacturer’s instructions.

### Data analysis

For all experiments, at least three independent experiments with technical triplicates were performed, and values were presented as mean±S.D.. Non-parametric statistic methods were applied. For two groups were compared using Mann-Whitney test, and for multiple comparisons were performed using Kruskal-Wallis test by GraphPad Prism. Differences were considered significant at * p<0.05, ** p<0.01 and *** p<0.001.

## Supporting information

S1 Fig atRAL and 4-HNE cause ARPE-19 cell death in dose- and time-dependent manners. atRAL and 4-HNE-induced ARPE-19 cell death was visualized and analyzed using PI staining and IncuCyte. (A) The represented pictures of atRAL or 4-HNE-induced ARPE-19 cell death with indicated concentrations at 24 h post-treatment. The dose and time responses of atRAL (B) and 4-HNE (C) induced-cell death were quantified by the number of PI positive cells. Each data point represents biological replicates (n = 3–4), and indicated as mean±S.D.
(TIF)

S2 Fig iPS-RPE has lower expression of apoptotic related genes compared to ARPE-19. mRNA from both ARPE-19 and iPS-RPE was collected and the expression levels of apoptotic related genes, such as CASP8, BAX, and FAS, were evaluated using qPCR. The relative expression levels were normalized to the relevant gene expression in ARPE-19. Each data point represents biological replicates (n = 3–4) and is indicated as mean±S.D. Statistical analysis was performed using the non-parametric Mann-Whitney test. ** p<0.01 and *** p<0.001, compared to the expression of the same gene in ARPE-19.
(TIF)
