# Supplementary figures and images for: Phenotypic high-throughput screening identifies aryl hydrocarbon receptor agonism as common inhibitor of toxin-induced retinal pigment epithelium cell death
Source: PLoS One. 2024 Apr 18;19(4):e0301239. doi: 10.1371/journal.pone.0301239 (PMC11025755; doi:10.1371/journal.pone.0301239)

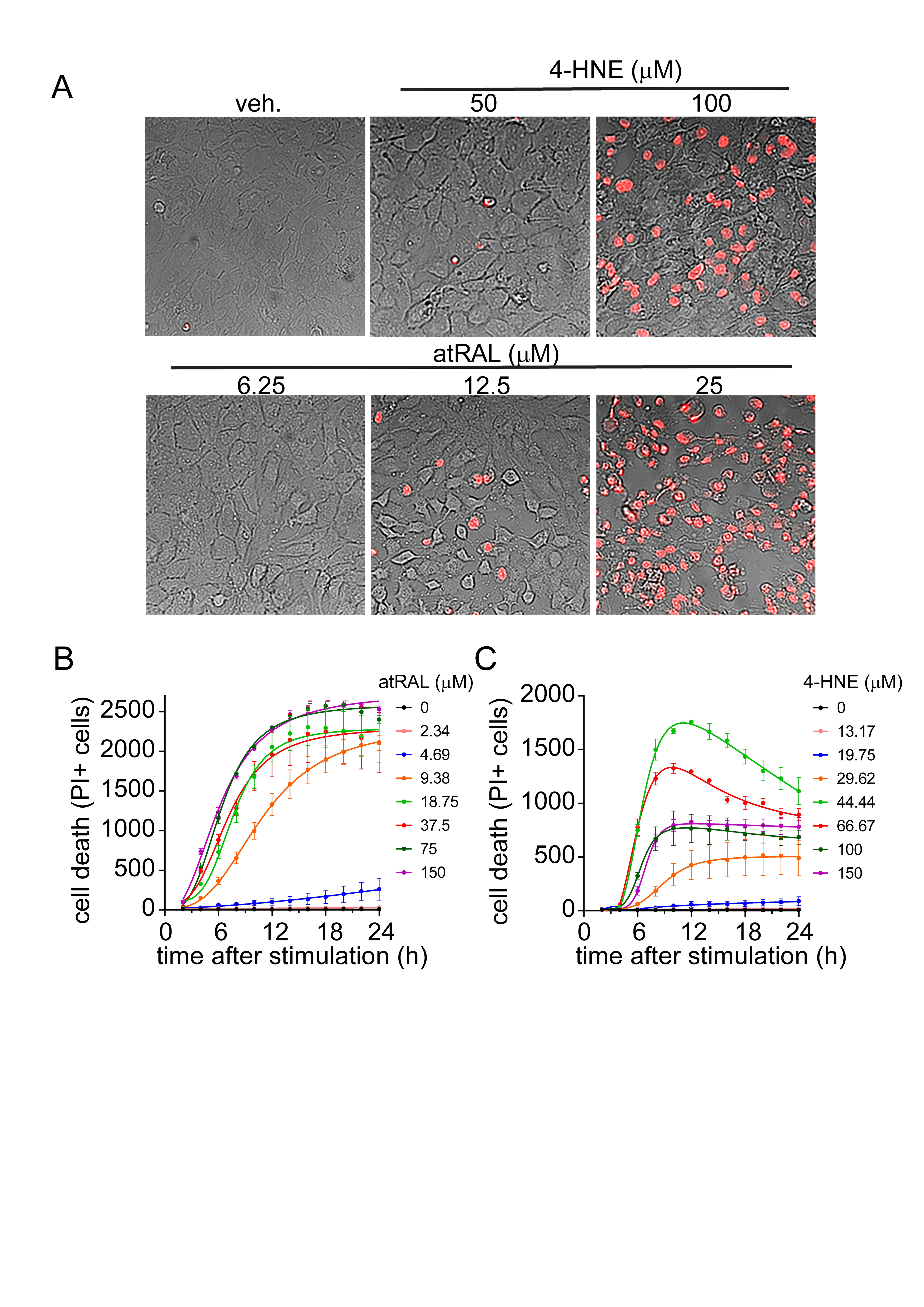

Supplement: S1 Fig — atRAL and 4-HNE-induced ARPE-19 cell death was visualized and analyzed using PI staining and IncuCyte. (A) The represented pictures of atRAL or 4-HNE-induced ARPE-19 cell death with indicated concentrations at 24 h post-treatment. The dose and time responses of atRAL (B) and 4-HNE (C) induced-cell death were quantified by the number of PI positive cells. Each data point represents biological replicates (n = 3–4), and indicated as mean±S.D. (TIF) [file pone.0301239.s001.tif]

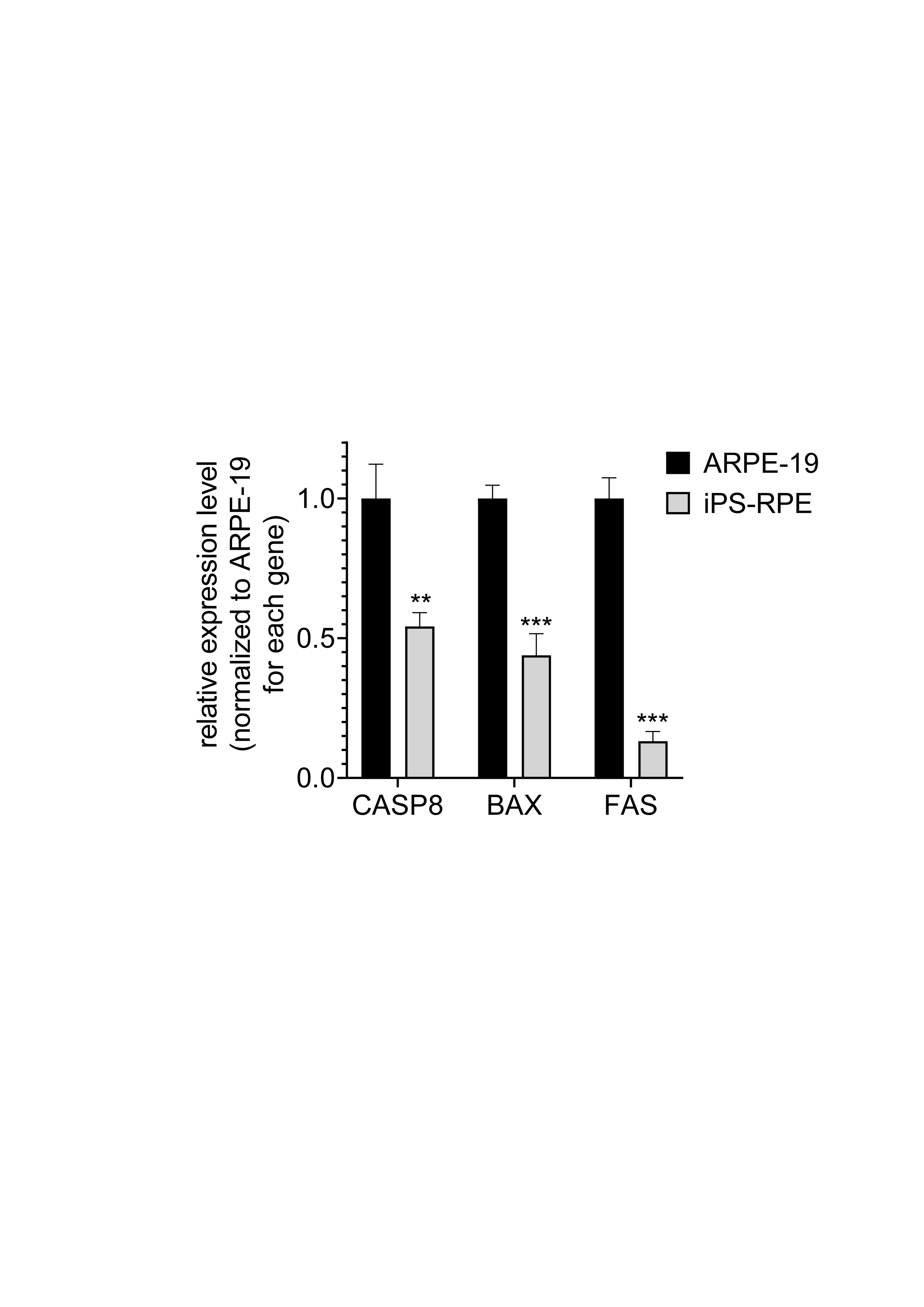

Supplement: S2 Fig — mRNA from both ARPE-19 and iPS-RPE was collected and the expression levels of apoptotic related genes, such as CASP8, BAX, and FAS, were evaluated using qPCR. The relative expression levels were normalized to the relevant gene expression in ARPE-19. Each data point represents biological replicates (n = 3–4) and is indicated as mean±S.D. Statistical analysis was performed using the non-parametric Mann-Whitney test. ** p<0.01 and *** p<0.001, compared to the expression of the same gene in ARPE-19. (TIF) [file pone.0301239.s002.tif]
